# Mr. Scammell's Case of Fracture

**Published:** 1807-08

**Authors:** Robert Scammell

**Affiliations:** Plymstock


### *To the Editors of the Medical and Physical Journal.*

GENTLEMEN,

ON Sunday, January 25, at twelve o'clock at night, a messenger came to me, to go to Turnchapel, in this neighbourhood, to render surgical assistance to a man who had met with a dreadful accident. On my arrival I understood he was a foreigner, a Calmuc Tartar, called Saphora, belonging to a Russian vessel the property of Signor Nicholas Panteli, then lying in the dock which belongs to Isaac Blackburne, Esq.; that he had been at a public-house, drinking with his companions, and had drank to such a degree as to produce the most complete intoxication. On his attempting to return on board, he passed over the planks laid from the edge of the dock to the ship's side; but in catching hold of a rope to ascend the ladder, he unfortunately fell backward, and was precipitated to a depth of thirty feet. In his fall, his head came in contact with a sort of step, of which there are several projecting from the sides of a dock, and are, I believe, technically called Altars. He was immediately taken up, and brought into his birth, in a state of perfect insensibility; indeed, even with scarcely any appearance of life. Whilst they were getting him up, he vomited repeatedly, and very copiously.

I found him stretched on a kind of bed, with every external appearance of an inanimate corpse, totally insensible to any question I put to him, with a pulse not to be distinguished; the tout ensemble strongly impressing my mind with the idea, that he had ceased to exist, and beyond the reach of any surgical assistance whatever. Too full of this belief, I was just ready to make a prognostic o that effect, when I was as suddenly struck with the propriety of a still more accurate examination. Often does it happen, that patients are pronounced to be without hope, and abandoned to their fate; indeed, the dreadful fiat, to a certainty, sometimes takes place, more from unafforded assistance than the violence of injury. I cannot help enforcing to the younger and inexperienced part of the profession, the absolute necessity, nay, the imperious call of humanity, never to abandon a patient to his fate, however dreadful the injury, however great may be the destruction of the organic system, or however the patient may be reduced by the action of disease; but let him consider, *Latet scintillula forsan.* The vis medicatrix naturæ of the older schools, or in whatever point of view such a principle may be understood in the modern, daily brings to our view such extraordinary facts as appear little short of the miraculous; whilst this vital principle has been known to overcome obstacles thrown in its way by the hand of officious ignorance. It has been very properly remarked,

Naturam expellas furcâ tamen usque recurret.

To return. On my requesting the people near him to turn him round, this slight motion caused instantly a copious vomiting, which though it showed that life had not entirely left him, yet pointed out that the system was most materially deranged. I soon found a most extensive laceration of the scalp; the occipito frontalis muscle was divided in a jagged manner, from where it becomes broad and tendinous, on the superior part of the cranium, down to its insertion into the orbicularis palpebrarum; this part was also completely denuded of its pericranium. On a still further examination, there appeared a fracture (of that description which has obtained the denomination of fissure) of the os frontis, extending from the sutura coronalis to the inferior portion of this bone, where it becomes the orbitar process, on the left side of the cranium. This fracture took an irregular zig-zag direction; it included both the lamellæ or tables of the cranium, there being an evident bloody secretion or discharge from the fissure, arising from the diploë, but which was rendered more certain by applying alternate pressure on each side of the fracture, where there was an evident elevation and depression of the bones, arising from the complete separation of both lamellæ. There was felt a considerable tumour at that part of the face where the levator labii superioris arises from the orbitar process of the superior maxillary bone.

The left clavicle was fractured near its articulation with the sternum. The third and fourth ribs were fractured, apparently, where the lesser pectoral muscle is inserted. The injury done to the pulmonary system was evident from the appearance of a widely diffused emphysema (of the propriety of this term I am not fully persuaded, though it is often used by Linnæus, Sauvagesius, &c.) over all that side of the thorax, marked by that peculiar crepitus which is evident to the ear as well as touch, plainly discovering that the spiculated point of the fractured rib had completely perforated the pleura, and entered the lungs.

The

The muscles of the right fore arm, on its inside, where they are covered by the aponeurotic expansion of the biceps, were much swollen, but there was no fracture either of the radius or ulna. An ecchymosis extended over the course of the Glutei muscles. A very careful examination convinced me that there was no depression of the fractured bone of the cranium, nor did I remark or suppose there were any evident symptoms of compression. It is generally understood that vomiting is an infallible sign of a compressed brain; but I have remarked, in several cases, that, like other dogmas, it is not without exception. I was aware, that the sinus which lays immediately in the inferior portion of the frontal bone, might be exposed to considerable injury, yet there was no symptom whatever absolutely indicative of extravasation of any kind, producing compression; consequently, I saw no necessity for the operation of the trephine, as I certainly consider it unnecessary where there are no symptoms sufficiently strong-marked to require its use.

After weighing carefully in my mind all the circumstances of this case, I was led to make this induction, that the man was *in articulo mortis,* and all assistance I could render him would be of no avail; still, I was determined to use every exertion in my power, whatever might be eventually the termination. I brought the lacerated, irregular edges of the scalp into contact, and retained them in that situation by the means of five sutures, and having spread the ungt. ceræ on some lint, applied it to the part affected, which was confined by a proper bandage, and over all a night-cap, as the best general mean of confining dressings, &c. to all wounds of the head. The fracture of the clavicle was reduced, and the necessary bandage applied. A broad roller was passed several times round the chest, &c. to prevent that distressing uneasiness which frequently arises from fractures of these bones during respiration, without this necessary precaution.

Bleeding has been, in general, considered injurious in cases of a concussion of the brain, as it has been said to produce sudden debility. However advisable it may be to follow general rules, yet in the sciences of medicine and surgery, deviations are often absolutely necessary, showing the too frequent inutility of classifications, and of too close an adherence to the systems of the Schools.

There were present in this case, general signs of a concussion of the sensorium; a largely dilated pupil, which had lost its contractile powers on opening the eye-lids to

 the the admission of an extraordinary degree of light; whilst the respiratory organs were not affected with laborious action, as is often the case in a state of compression, but performed their office in a slow, yet almost imperceptible manner. I considered it necessary to attempt to bleed him, and made a considerable large orifice, but scarcely a drop of blood followed the lancet. I enlarged the orifice, but to no purpose. This did not tend to diminish my apprehensions for the event.

I directed, that in case of his surviving, he should be kept as quiet as possible; should take no solid nutriment whatever, but drink an infusion of balm, or water-gruel; and above every thing, to admit no stimulus from light, particularly that of a candle. I then left him, strongly prepossessed with the idea that his existence would be but for a very short time.

26th. On my arrival this morning, to my great surprise, I found him still living. He lay in a comatose state the whole of last night; his pulse was perceptible, yet unusually slow; the respiration enfeebled, and at long intervals. On my stimulating the system, purposely to try its effect on the sensorium, he gave some signs of returning sensation. I directed him to be still kept perfectly quiet, to continue the same diluting liquids, and gave him a solution of magnesia vitriolata. It may not be improper to remark, that I have found this medicine peculiarly efficacious in stomachic irritability, where every thing has been instantly ejected; in small doses, it has taken off this irritation: In two cases, where it was absolutely necessary the stomach should retain a certain laxative medicine, it was only effected by its combination with ℥jß of the vitriolated magnesia. The dressings to his head to remain as at first applied.

27th. He passed a night without any sleep; appearances are more favourable; pulse and respiration becoming now more natural. The laxative medicine operated well. To continue his diluting liquids; as I had greatly to apprehend the re-action of the system, which might proceed to such a length, as to produce inflammation and a train or subsequent distressing circumstances.

28th. This morning every symptom more favorable; no sleep last night, but he was enabled in some measure, to explain his feelings; he complained most severely of an unextinguishable thirst, and a most distressing degree of pervigilium. The former symptoms, I have before remarked, in an evident concussion of the brain, and to as great a degree; but as I am at a loss to explain the cause

I shall

I shall not attempt it. To endeavour to overcome this morbid propensity, his drink was occasionally acidulated. To day I examined the state of the scalp, and found it almost entirely healed by that desirable process, the first intention, except in two small parts; the one at the internal canthus, the other near where the corrugator supercilii muscle is inserted into a portion of the frontalis, and rather extending to the superciliary ridge; here the pus was of a most healthy appearance. The eye was uninjured, as he could discover minute objects as well as ever. A pledget, spread with the ungt. ceræ, was again applied; I gave him this evening the tinct. opii. gtt. xxx. and permitted him to eat a little soft crumb of bread with his water gruel; bowels regular as to evacuations.

29th. The pervigilium and thirst still tormented him to an almost insufferable degree; in other respects every symptom tending to amendment. The other injured parts of his body gave him but little uneasiness; he now complained of very violent pain at the hind part of the head, between the occiput and the second vertebra of the neck; no doubt, from the great injury and consequent violent stretching of the ligaments which connect the *processus dentatus* of the second vertebra to the occipital bone; it was increased to a very violent degree, particularly by the least rotatory motion. I increased the dose of the tinct opii. to gtt. L.; care being taken to preserve a regularity of intestinal evacuation by the use of the vitriolated magnesia.

30th. The thirst and watchfulness still his great sources of complaint; in other respects much better. The scalp nearly quite healed; the ligatures sloughing away; he is to take food a little more nutritious, but with the greatest caution as to quality or quantity. As the opium produced no sort of uneasiness or bad effect, I judged it proper to increase the dose. to gtt. lx.

31st. Had a little sleep last night; his thirst was not so great; the ligatures all came away; the bowels too constipated. To take the saline solution, as before, according to circumstances. The pain at the back part of the neck gives him still greater uneasiness, yet unattended with tension, or any sort of particular local appearance.

To increase carefully his sustenance.

Feb. 1st. Gradually proceeding to convalescence; the scalp perfectly healed; he is able to sit up in his bed; slept a little last night without his opium.

From this time his recovery was rapid, and on the 6th he was sufficiently well to follow me from his birth to the

 captain's captain's cabin, being the twelfth day from his receiving the injury. It is to be remarked, that he felt some pain in his neck a few days beyond this period; but it then entirely left him, and he has since been able to do his duty as a seaman, in every respect, without the slightest inconvenience.

I think this case should carry that degree of conviction with it, never to abandon a patient, though he may be in extremes; and that as long as life exists, hopes should still encourage us. It also proves the strength of the *vis vitæ*, or of Nature, in her struggles to overcome the effects of disease; these efforts are in no instances, more visible than in injuries received from external violence, as passing immediately under the cognizance of the more sensible faculties; not but the activity of the constitution is equally exerted in subduing the malignancy of contagion, but here the *nisus* is more obscure, and consequently less observed.

That Nature is to be implicitly followed, is what I would by no means infer; she has her *ne plus ultra*, and very often requires the assistance of art; as, for instance, in that soft and spongy kind of granulation, the Υπερσαρκωσις of ulcers, which frequently demands the repeated application of powerful escharotics. Again, we have many, too many fatal instances, where Nature as soon carries on the process of suppuration in the lungs to the certain destruction of the unhappy sufferer, as she would in the cellular membrane of an extremity, without any hazard to the constitution. Yet the absolute uncertainty of forming a true prognosis, as to the event of accidents, requiring the interposition of surgery, is a true but lamentable fact, as I have elsewhere remarked. As decided proofs, I would at this time briefly observe, that some years since I was concerned in a case, where a man, from a slight cut in his hand, was seized with tetanus, which killed him; whilst, very lately, another had his finger nearly divided, with loss of substance and exposure of the joint, who was perfectly cured. A man, from a lacerated wound in the extremity, died a few days after; whilst another, with a wound in the same part, extending half its circumference, with a protrusion of the tibia, to the extent of three inches, has perfectly recovered.

The most acute powers of discrimination are necessary to enable us to form any kind of judgment in those cases: we should ever recollect, that there are undoubtedly many excellent excellent rules laid down by systematic writers, to direct it; but, above all, we should have particular regard,

1st. To the season of the year.

2d. Whether a proper and continued attention can be paid, during the necessary confinement.

3d. The situation, &c. of the injury.

4th. Whether inflicted by a sharp instrument, or crushed by a heavy compressing power.

5th. The age of the patient.

6th. Though last, by no means the least, the predisposition to disease, or, in plain terms, the habit of body.

I am, &c.

*Plymstock, June* 10, 1807. ROBERT SCAMMELL.

